# Long-term Proton Pump Inhibitor Administration Caused Physiological and Microbiota Changes in Rats

**DOI:** 10.1038/s41598-020-57612-8

**Published:** 2020-01-21

**Authors:** Yu-Chen S. H. Yang, Hsuen-Wen Chang, I-Hsuan Lin, Li-Nien Chien, Min-Ju Wu, Yun-Ru Liu, Peiguo G. Chu, Guoxiang Xie, Fangcong Dong, Wei Jia, Vincent H. S. Chang, Yun Yen

**Affiliations:** 1 Joint Biobank, Office of Human Research, Taipei Medical University, Taipei, Taiwan; 2 Laboratory Animal Center, Office of Research and Development, Taipei Medical University, Taipei, Taiwan; 3 TMU Research Center of Cancer Translational Medicine, Taipei Medical University, Taipei, Taiwan; 4 School of Health Care Administration, College of Management, Taipei Medical University, Taipei, Taiwan; 5 Department of Physiology, School of Medicine, College of Medicine, Taipei Medical University, Taipei, Taiwan; 6 Department of Pathology, City of Hope National Medical Center, Duarte, CA 91010 USA; 7 University of Hawaii Cancer Center, Honolulu, Hawaii 96815 USA; 8 The PhD Program for Cancer Biology and Drug Discovery, College of Medical Science and Technology, Taipei Medical University, Taipei, Taiwan; 9 Division of Chemistry and Chemical Engineering, California Institute of Technology, Pasadena, CA 91125 USA; 10 Graduate Institute of Cancer Biology and Drug Discovery, Taipei Medical University, Taipei, Taiwan; 11 Cancer Center, Taipei Municipal WanFang Hospital, Taipei, Taiwan

**Keywords:** Microbial communities, Experimental models of disease

## Abstract

Proton pump inhibitors (PPIs) are used for the long-term treatment of gastroesophageal disorders and the non-prescription medicines for acid reflux. However, there is growing concerns about PPI misuse, overuse and abuse. This study aimed to develop an animal model to examine the effects of long-term use of PPI *in vivo*. Twenty one Wistar rats were given omeprazole orally or intravenously for 30 days, and caerulein as a positive control. After euthanization, the serum and stool were collected to perform MS-based quantitative analysis of metabolites. We carried out 16S-based profiling of fecal microbiota, assessed the expression of bile acid metabolism regulators and examined the immunopathological characteristics of bile ducts. After long-term PPI exposure, the fecal microbial profile was altered and showed similarity to those observed in high-fat diet studies. The concentrations of several metabolites were also changed in various specimens. Surprisingly, morphological changes were observed in the bile duct, including ductal epithelial proliferation, micropapillary growth of biliary epithelium, focal bile duct stricture formation and bile duct obstruction. These are characteristics of precancerous lesions of bile duct. FXR and RXRα expressions were significantly reduced, which were similar to that observed in cholangiocarcinoma in TCGA and Oncomine databases. We established a novel animal model to examine the effects of long-term use of omeprazole. The gut microbes and metabolic change are consequences of long-term PPI exposure. And the results showed the environment *in vivo* tends to a high-fat diet. More importantly, we observed biliary epithelial hyperplasia, which is an indicator of a high-fat diet.

## Introduction

Proton Pump Inhibitors (PPIs) are one of the most prescribed drugs in many countries. It inhibits the H + /K + ATPase enzyme system in the stomach to suppress acid production, resulting in higher gastric pH. Long-term PPI use is an effective treatment for gastroesophageal disorders and *Helicobacter pylori* infection, and are taken without prescription to relieve heartburn^1^. Despite their increased usage and popularity, over-the-counter (OTC) PPIs such as omeprazole, lansoprazole and pantoprazole are prone to overuse and misuse. There are growing concerns in the medical community about the inappropriate and overuse of PPIs^2–4^.

Long-term PPI use have been associated with adverse consequences, including chronic kidney injury, acute kidney injury, acute interstitial nephritis, hypomagnesemia, *Clostridium difficile* infection, community-acquired pneumonia, bone fracture and increased risks of gastric and periampullary cancers development and death^5–8^. Many of these are retrospective and longitudinal observational studies, therefore bias may be introduced and results can be influenced by confounding variables. Furthermore, the mechanisms behind the reported adverse effects remain unclear^9,10^.

PPI users tend to have a less healthy gut microbiome than non-users, with significant increase of *Enterococcus*, *Streptococcus*, *Staphylococcus* and *Escherichia coli*^11^. PPIs have been shown to cause changes in bile salt composition in Barrett’s esophagus patient^12^. In view of these, we postulated that chronic PPI use can lead to the build-up of unhealthy gut microflora and disrupts normal gallbladder and biliary functions to induce biliary tract diseases. In this study, we investigated the effects of long-term omeprazole exposure on fecal microbiome and pathophysiology of Wistar rats and discussed the possibility of promoting physiological change.

## Results

### Animal model of Omeprazole long-term treatment

**Figure 1:**
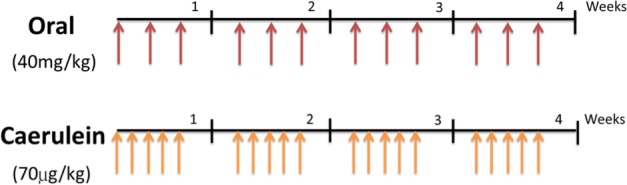
Animal model of long-term omeprazole treatment. Schedule of drug administration in Wistar rats in 30 days. Rats were treated with caerulein (positive control) 5 times per week (total 20 doses), omeprazole was administered orally 3 times per week (total 12 doses). Saline (negative control) was administered as a negative control.

In this study, we used a rat model to assess the effects of long-term omeprazole exposure to living organisms. In previous reports, caerulein (an analogue of the human gastrointestinal hormone cholecystokinin) was used to stimulate smooth muscle contraction and release of biliary and pancreatic enzymes. At high concentrations, caerulein is reported to stimulate the inflammation, acinar loss, and fibrosis typically found in pancreatitis^13,14^. We therefore adopted caerulein treatment (70 μg/kg) as a positive control. Omeprazole was administered orally (40 mg/kg) for 30 days. Intravenous saline was used as a negative control (Fig. 1).

### Omeprazole caused fecal bacterial changes in rats

**Figure 2:**
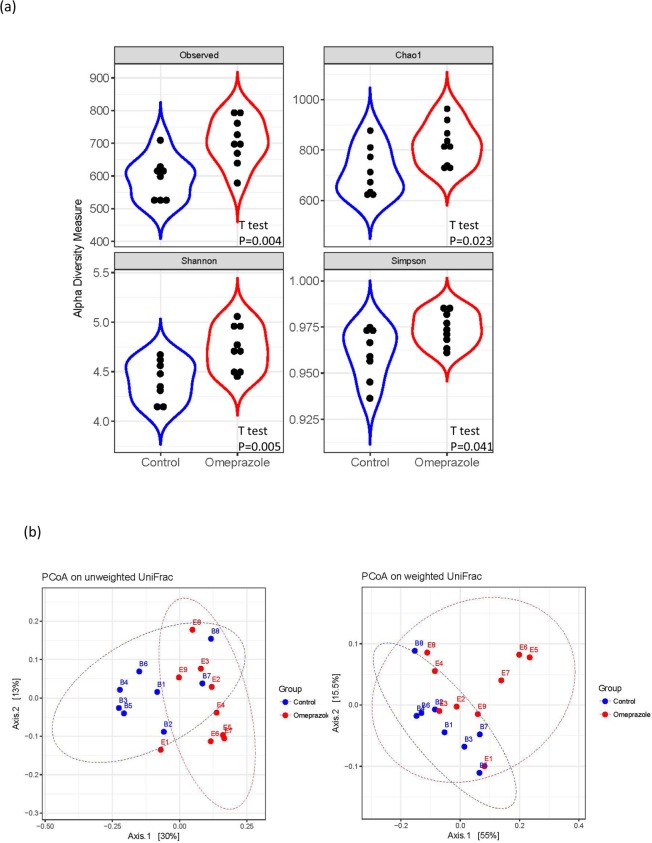
Fecal microbiome distribution in rats with long-term omeprazole treatment. The feces of rats treated with and without omeprazole for 30 days were prepared for fecal microbiome profiling by high-throughput sequencing of the 16s rRNA gene with the Illumina MiSeq system. (**a**) Alpha-diversity of omeprazole treated samples and untreated controls. Statistical comparison between two groups was performed with exact Wilcoxon-Mann-Whitney test and significant differences were obtained for all four indices (at α = 0.05) (**b**) Principal coordinate analysis (PCoA) plot based on Unweighted or Weight UniFrac distance of omeprazole treated samples and untreated controls. Significant difference in beta-diversity was evaluated with permutational multivariate analysis of variance (vegan::adonis, 1000 permutations) and beta-dispersion was quantified with betadisper (vegan::betadisper, 1000 permutations). Both indices achieved adonis P < 0.05 and betadisper P > 0.05.

**Figure 3:**
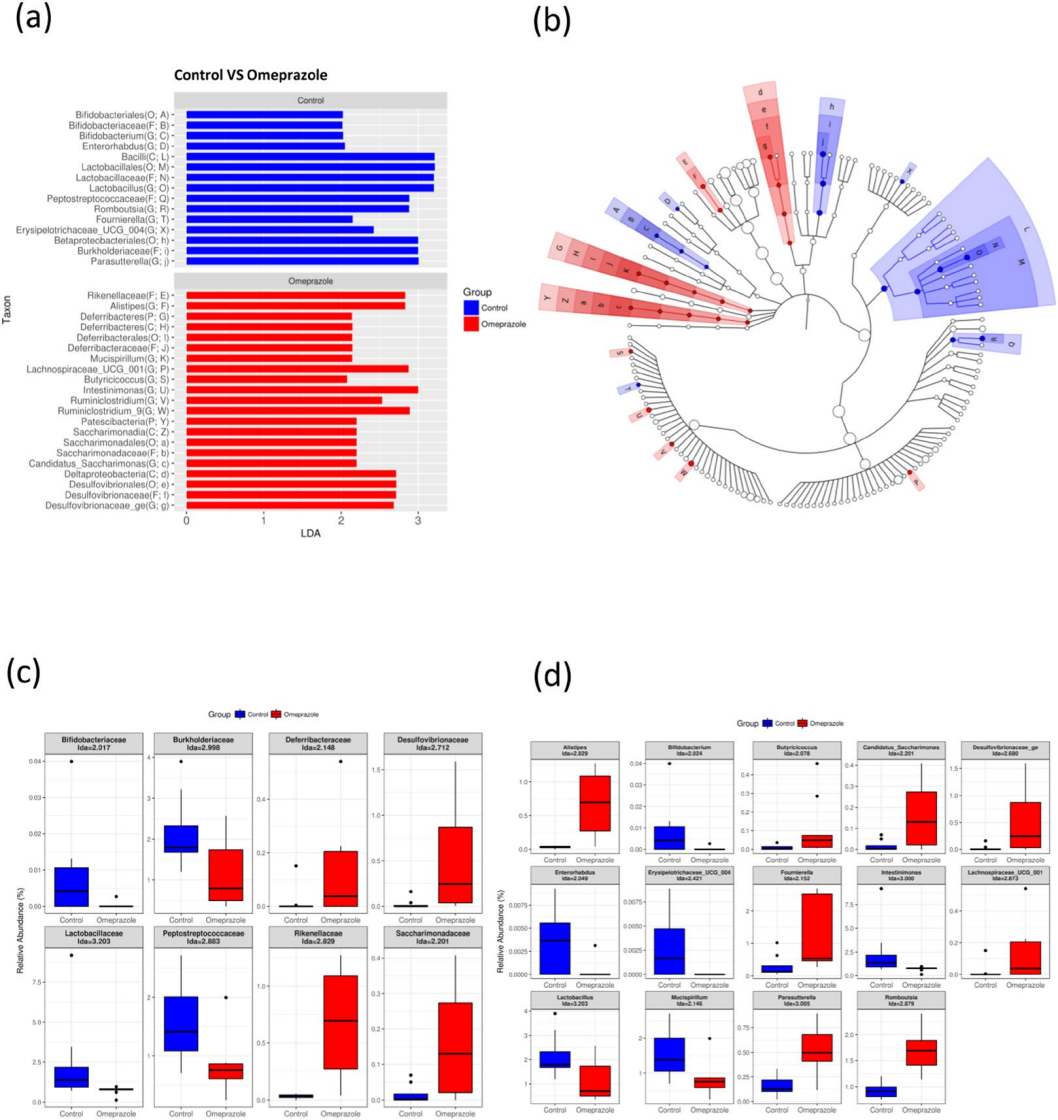
Gut microbiota is changed in rats with long-term omeprazole treatment. Linear discriminant analysis (LDA) effect size (LEfSe) analysis of gut microbiota changes in rats with long-term omeprazole treatment. Significant biomarkers were defined as taxa with a LDA score (log10) ≥ 2. (**b**) Significant taxa were highlighted on the cladogram. P: Phylum; C: Class; O: Order; F: Family; G: Genus. Bacteria at (**c**) family level and (**d**) genus-level with significant changes in abundance with omeprazole treatment (LDA ≥2).

Gut microbiome shows that long-term omeprazole used leads to an increase in alpha-diversity(Fig. 2a). Unweighted and weighted UniFrac PCoA analysis also showed omeprazole-treated rats displayed a different fecal microbiome profile compared to the untreated rats (Fig. 2b). Although there was no statistically significant difference in microbial composition at the phylum level (Fig. 3 and Table 1), there was a notable increase in the Firmicutes/Bacteroidetes ratio in the omeprazole-treated rats (Table 1). Also, significant changes at the family level including Bifidobacteriaceae, Lactobacillaceae, Peptostreptococcaceae, Burkholderiaceae, Rikenellaceae, Deferribacteraceae, Saccharimonadaceae and Desulfovibrionaceae, and at genus level including *Bifidobacterium*, *Enterorhabdus*, *Lavtobacillus*, *Romboutsia*, *Fournierella*, *Erysipelotrichaceae_UCG_004*, *Parasutterella*, Alistipes, Mucispirillum, *Lachnospiraceae_UCG_001, Butyricicoccus, Intestinimonas, Ruminiclostridium, Ruminiclostridium_9, Candidatus_Saccharimonas* and *Desulfovibrionaceae* were identified in omeprazole-treated rats (Fig. 3).

**Table 1:** Bacterial phyla identified from the fecal microbiome of rats treated with omeprazole and control.

Phylum	Control		Omeprazole		U-test
	Mean^a^	SD^b^	Mean	SD	
Actinobacteria	0.001	0.000	0.000	0.000	0.11
Bacteroidetes	0.551	0.095	0.467	0.118	0.17
Epsilonbacteraeota	0.008	0.022	0.012	0.028	0.27
Firmicutes	0.417	0.109	0.499	0.121	0.17
Proteobacteria	0.023	0.009	0.019	0.009	0.96
Firmicutes/Bacteroidetes Ratio	0.757		1.069		

^a^Average proportion of identified phylum.

^b^Standard deviation.

### Omeprazole-induced metabolites changes in rat

**Figure 4:**
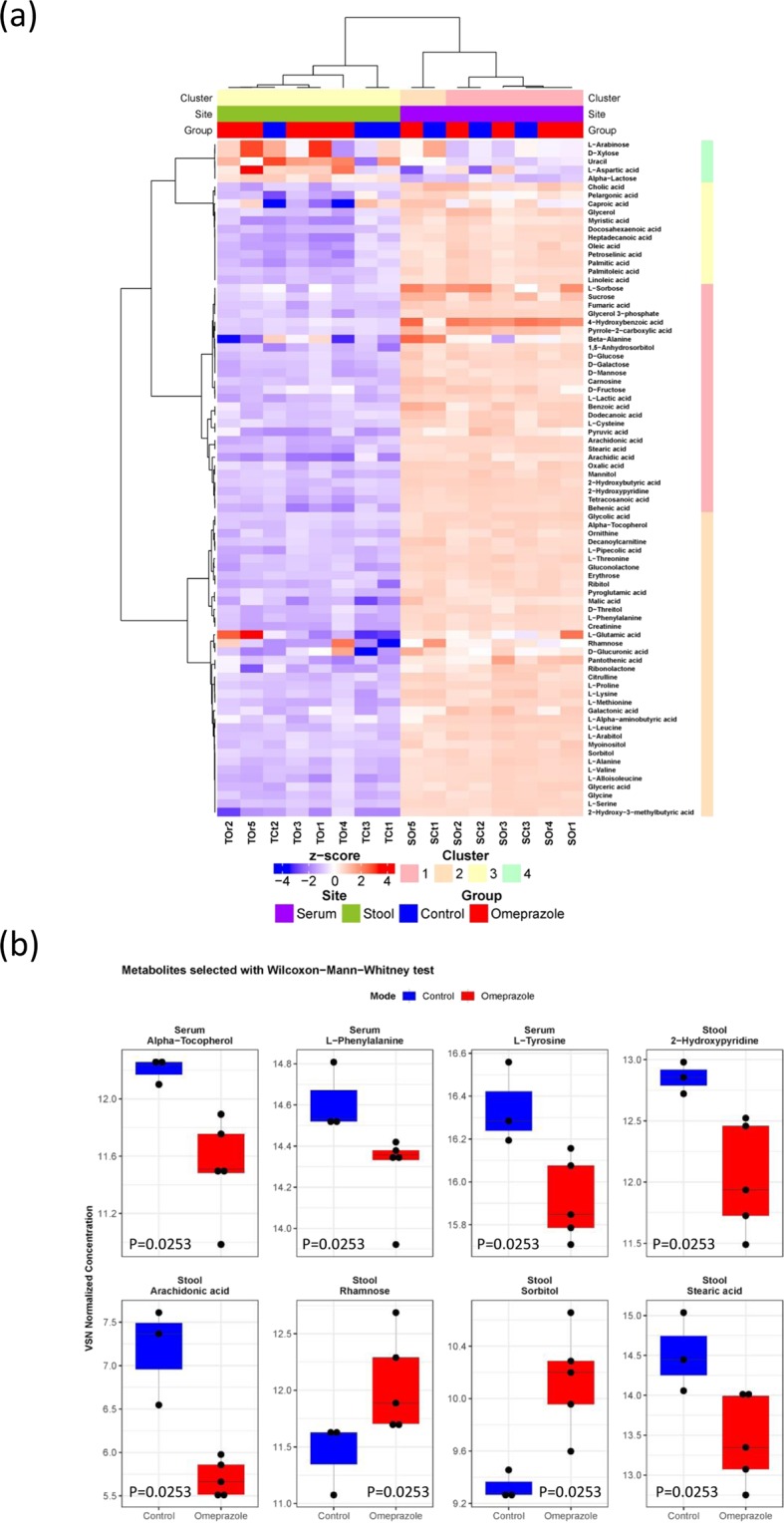
Comparison of metabolites between omeprazole-treated and control Wistar rats. Rats were sacrificed at the end of the experiment (day 30) and serum and stool were collected. The serum and stool metabolites were analyzed by GC-TOFMS. (**a**) Heatmap showing the abundance of the identified metabolites (**b**) Statistical comparison between treated and control rats was performed with exact Wilcoxon-Mann-Whitney test at α = 0.05. Three and five metabolites were found to show significant differences in serum and stool samples respectively.

In this study, we examined the composition of metabolites isolated from serum and stools of rats after 30 days of omeprazole treatment. Several metabolites in serum and stool showed significant changes in concentrations. In rat serum, α-tocopherol, L-phenylalanine and L-tyrosine were lower in the omeprazole-treated group. In stool, arachidonic acid and stearic acid concentration were significantly lower in the omeprazole-treated group, whereas rhamnose and sorbitol were significantly higher (Fig. 4). These metabolites achieved a statistical significance of P < 0.05, however they did not pass the FDR threshold of 5%.

### Omeprazole-induced neoplastic transformation of bile duct tissue

**Figure 5:**
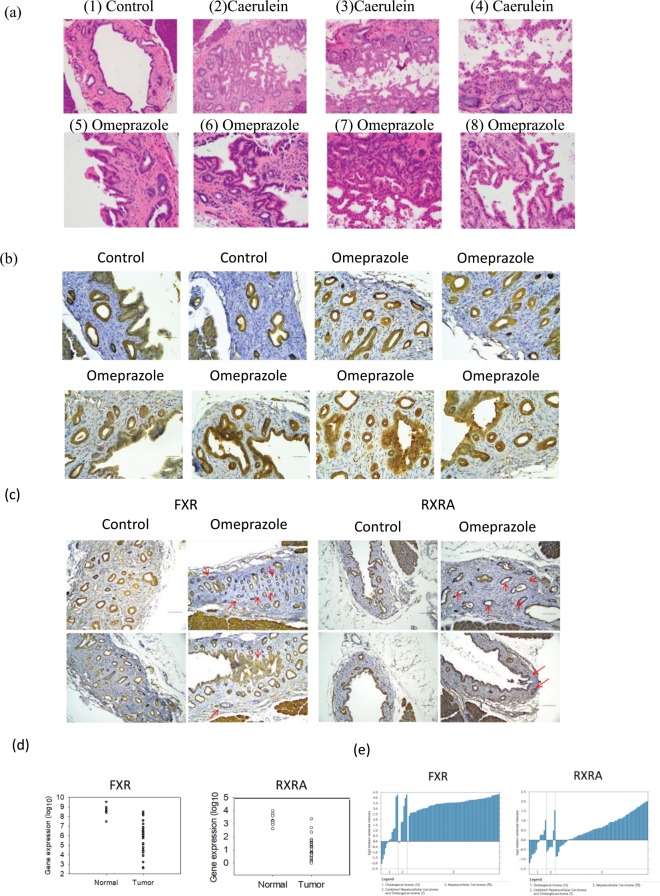
Omeprazole induced lesions in the bile duct. The animals were sacrificed after 30 days. Bile ducts were removed and washed with iced phosphate buffer solutions. The tissues were embedded in paraffin wax (**a**) HE stain and (**b**) stained with anti-CK-19 antibody and examined. The bile ducts of rat treated with PPIs display morphological distortion and thickening of the bile duct epithelium. (**c**) Stained with anti-FXR and RXRα antibodies. (**d**) RNA expression levels of *FXR* and *RXRα* in cholangiocarcinoma and normal liver obtained from the TCGA database. (**e**) The Oncomine™ (Compendia Bioscience, Ann Arbor, MI) database (http://www.oncomine.org/) was used to compare relative gene expression levels of *FXR* and *RXRα* in (1) cholangiocarcinoma, (2) combined cholangiocarcinoma and hepatocellular carcinoma, and (3) hepatocellular carcinoma.

In this study, the bile duct epithelium of rats treated with caerulein (Fig. 5a-2, -3 and -4) and omeprazole (Fig. 5a-5, -6, -7 and -8) showed marked ductal epithelial proliferation. The pseudostratification of bile duct epithelium as well as micro papillary growth patterns resembled those of adenocarcinoma^15^. Ductal proliferating cells also revealed mild to moderate cytological atypia and increased mitotic activity. Morphological changes in the omeprazole group were confirmed by IHC analysis using keratin 19 (CK-19), a known marker for gastroenteropancreatic and hepatobiliary tumors^16^. The CK-19 staining results showed rats treated with omeprazole developed focal bile duct strictures and bile duct obstruction (Fig. 5b).

Additional IHC analysis was performed on FXR and RXRα, which are the master transcriptional regulators of bile acid metabolism and has a protective role in carcinogenesis. FXR and RXRα play important roles in the malignancy of several cancers, including CCA^17^. In our study, staining using anti-FXR and anti-RXRα antibodies revealed that both FXR and RXRα were down-regulated in the bile duct of rats treated with omeprazole compared to control (Fig. 5c). Decreased *FXR* and *RXRα* RNA expression was also found in human cholangiocarcinoma (CHOL) dataset in the TCGA database (Fig. 5d). In relation to other hepatobiliary cancers listed in the Oncomine database, cholangiocarcinoma had the lowest *FXR* and *RXRα* RNA expression when compared to the combined hepatocellular carcinoma and cholangiocarcinoma and also the hepatocellular carcinoma cases in the Woo Liver dataset (Fig. 5e).

## Discussion

Since the emergence of PPI in the 1970’s, it has been widely used for the treatment of a variety of gastric acid-related diseases. In recent years, focus on the adverse side effects of PPIs has gained growing concerns. Common side effects from taking to PPIs include headache, diarrhea, constipation, abdominal pain, flatulence nausea and rash^18^. In relation to cancer, only PPI-associated hypergastrinemia has been directly linked to gastric cancer^19^.

In our study, the 30-day regimen in the 6-month laboratory rat is equivalent to approximately 3 years of exposure in human years^20^. Ductal epithelial atypia and cribriform and micropapillary growth patterns were also observed in bile duct epithelium under long-term exposure to omeprazole in Wistar rats. The proliferating ductal cells showed mild to moderate cytological atypia, similar to bile ductal dysplasia in cholangiocarcinoma(CCA) patients^21^. These morphological abnormalities were also found in the positive control group taking caerulean, while absent in negative control (saline). Interestingly, this biliary epithelial hyperplasia also observed on C57Bl/6 J mice with a high-fat diet^22^. This means that the phenomenon caused by omeprazole in rats is related to the high-fat diet.

Omeprazole exposure affects gut microbiota and influences the composition similar to that observed in high-fat-diet animal models. The Firmicutes/Bacteroidetes ratio, which is a marker of high-fat diet, were increased in the omeprazole-treated group (Table 1)^23^. The Bifidobacteriaceae, Lactobacillaceae and Peptostreptococcaceae decreased abundance, *Intestinimonas*, Desulfovibrionaceae, Deferribacteraceae, *Lachnospiraceae* and *Candidatus_Saccharimonas* showed increase in abundance in omeprazole-treated rats (Fig. 3), similar changes were also observed in high-fat diet model^24–26^. Interestingly, such gut microbiota composition was also similar to that observed in cancer. Bifidobacteriaceae, Lactobacillales and *Erysipelotrichaceae* were reported to be reduced in CRC patients, and *Lachnospiraceace*, Rikenellaceae, *Alistipes* and Desulfovibrionaceae were increased in CRC patients^27–30^.

Interestingly, our global metabolite profiling identified unique metabolic changes in rats exposed to omeprazole. Specifically, we found significant lower concentrations of α-tocopherol, L-phenylalanine and L-tyrosine in omeprazole-treated rat serum. α-tocopherol is a fat-soluble vitamin E with potent antioxidant activity and is involved lipid metabolism^31^, and L-phenylalanine and L-tyrosine are aromatic amino acids and have been shown to decrease in high-fat diet mouse model^32^. In stool, the 20-carbon chain unsaturated fatty acid arachidonic acid and 18-carbon chain saturated fatty acid stearic acid were decreased in omeprazole-treated rat, both fatty acids are involved in lipid metabolism and biosynthesis of unsaturated fatty acid. Rhamnose (a 6-carbon deoxy-sugar) and sorbitol (six-carbon sugar alcohol) were elevated in omeprazole-treated rat and are involved in fructose and mannose metabolism^33,34^. These changes have been linked to high-fat diet-induced metabolism. Recent researches have highlighted the increased risks of multiple cancers with high-fat diets, including CCA^35–37^. Further studies are warranted to clarify the exact mechanism by which omeprazole can induce precancerous lesions in the bile duct.

CCA is the second most frequent primary hepatic malignancy, originating from biliary epithelial cells. CCA is known to develop through a multi-step progression^38^. This process typically starts with epithelial hyperplasia leading to dysplasia and eventually adenocarcinoma. CCA patients often do not respond well to conventional chemotherapy and radiotherapy, surgical resection remains the only effective therapy for early stage tumors^39^. Therefore it remains urgent to identify the molecular mechanisms underlying the development and progression of CCA for preventive and therapeutic purposes. In 2016, Chien *et al*. reported a dose-dependent association between PPI use and risk of periampullary cancers, including CCA^6^. The precancerous lesions we observed in rats under chronic PPI exposure provide further evidence to highlight the risk of long-term overuse of PPI. Gut microbiota changes in CCA patients and the effect of PPIs induced gut microbiome change had been independently studied but never concurrently. We believe our study is first of its kind to report the association between long-term PPI use, changes in fecal microbiota and neoplastic transformation of biliary tissues. Microbiome analysis revealed significant imbalances in the fecal flora of rats exposed to PPIs compared to control. These results suggest that the gastric and gut microbiota was affected by the change in gastric pH and the symbiotic relationship was disrupted. Microorganisms and the surrounding tissue microenvironment can cross-talk and influence each other to contribute to cancer. Future investigations on how alterations in gut microbiome impact the development of CCA will help the development of strategies for CCA treatment and prevention.

We performed IHC staining to compare the FXR and RXRα protein expression in rat bile duct exposed to omeprazole versus control. The decrease in FXR and RXRα expressions observed in this study was in accordance with human cholangiocarcinoma datasets from Oncomine and TCGA. Interestingly, loss of FXR protects diabetic mice against diet-induced or genetic obesity and accelerates liver carcinogenesis^40^. In our animal models, the body weights of PPI-treated rats also remained unchanged compared with controls (Supplementary Fig. 1). Hence, the onset of early biliary tract abnormalities is difficult to observe.

It is well established that the gut microbiota has profound effects on bile acid metabolism, host metabolism and human health^41,42^. Imbalances to microbiome environment and bile acids may play important role in tumorigenesis of CCA^41,42^. Regulation of this delicate balance as well as FXR/RXRα might provide promising therapeutic approaches to treating CCA patients.

We have established a novel animal model to observe the effects of long-term omeprazole use *in vivo* and discuss the potential risks of cholangiopathy. Overall, our results provide evidence that PPIs may be a risk factor for CCA development. In our design, the duration of PPI exposure is equivalent to 3 human years. We wish to emphasize that PPI should be use following proper treatment and deprescribing guidelines, and our work highlights the need for increase awareness of physicians, patients and general public potential risks associating with PPI misuse and overuse.

## Materials and Methods

### Animal study

A total of 21 Wistar rats were purchased from NARLabs (National Applied Research Laboratories, Taiwan). All animals were maintained and handled in an accredited facility in accordance with IACUC (Institutional Animal Care and Use Committee) guidelines and the animal study protocol was approved by the Affidavit of Approval of Animal Use protocol, Taipei Medical University (License NO:LAC-2016-0352). A long-term (30 days) experiment was designed. At six weeks of age, rats were randomly assigned to one of four exposure groups: (1) oral omeprazole (40 mg/kg) (SIGMA-ALDRICH, St Louis, MO) (2) saline as negative control group; or (3) caerulein (20 μg/kg) as a positive control group. All animals were sacrificed on day 30 upon completion of their designated schedule. Rat bile ducts were harvested and washed with ice-cold phosphate buffered solutions for IHC analysis. Tissues were also embedded in paraffin wax for histological and immunological assays.

### Immunohistochemistry

IHC performed as previously described^43,44^. In brief, sections were cut onto adhesive-coated glass slides at 4-µm thickness, de-waxed, re-hydrated with PBS, and followed by incubation with anti-farnesoid X receptor (FXR) (GTX113867: GENETEX, Hsinchu City, Taiwan) and anti-retinoid X receptor-α (RXRα) (GTX113829: GENETEX) antibodies. Immune complexes were detected using the ChemMate DAKO EnVision kit (K5001; DAKO, Carpinteria, CA), and then the slides were counter-stained with hematoxylin. A negative control was simultaneously performed by incubating the corresponding tissue sections in a solution without the primary antibody.

### 16S rRNA gene sequencing and NGS analysis

The stool of rats were collected at day 30, and purified by QIAamp Fast DNA Stool Mini Kit (QIAGEN, Germany). The library preparation follows the protocol of 16S Ribosomal RNA Gene Amplicons for the Illumina MiSeq System. Sequence reads have been deposited in the European Nucleotide Archive (ENA) under accession number PRJEB28574.

The universal primers (341 F and 805 R) that were used to amplify the V3-V4 region of the bacterial 16S rRNA genes were first removed from the demultiplexed, paired reads using cutadapt (v 1.12; DOI:10.14806/ej.17.1.200). The filtered reads were processed in the R environment (v 3.3.3) using R package DADA2 (v 1.3.5)^45^ following the workflow described in Callahan *et al*.^46^ without performing rarefying procedure. Briefly, the forward and reversed reads were filtered and trimmed based on the read quality score and read length. Dereplication was then performed to merge identical reads, then reads were subjected to the denoise DADA2 algorithm which alternate between error-rate estimation and sample composition inference until they converge on a jointly consistent solution. Finally, the paired reads were merged that required a minimal of 20 bp overlap and chimeras were subsequently removed. At this point, we obtained a list of V3-V4 sequence variants (SVs) found in our samples that were inferred by DADA2, as well as the frequency of each SV in each sample. Taxonomy assignment was performed using the SILVA database (v128)^47^ as the reference with a minimum bootstrap confidence of 80. Multiple sequence alignment of the SVs was performed with DECIPHER (v2.2.0) and phylogenetic tree was constructed from the alignment using phangorn (v2.2.0)^48^. The count table, taxonomy assignment results and phylogenetic tree were consolidated into a phyloseq object, and community analyses were performed using phyloseq (v1.19.1)^49^. The alpha-diversity indices were calculated using the estimate_richness function from the phyloseq package. Statistical comparison between treatment and control was performed with exact Wilcoxon-Mann-Whitney test (at α = 0.05). UniFrac distances were calculated using the GUniFrac package (v1.1) to assess the community dissimilarity between groups^50^. Principal coordinate analysis (PCoA) ordination on UniFrac distances was performed and the adonis and betadisper functions from the vegan package (v2.4; https://CRAN.R-project.org/package = vegan) were used to conduct statistical analysis for the dissimilarity of composition among groups and the homogeneity of dispersion respectively. Microbiota enrichment analysis between groups was carried out by using the Linear Discriminant Analysis (LDA) Effect Size (LEfSe) method with alpha set at 0.05 (Kruskal-Wallis and Wilcoxon tests) and logarithmic LDA score of 2 or more^51^ and visualized as cladogram by using GraPhlAn^52^

### Global metabolite profiling by GC-TOFMS

For each animal, serum and stool were removed, divided into small portions, flash-frozen in liquid nitrogen, and stored at −80 °C until sample preparation and analysis. Standards for approximately 1,000 mammalian metabolites were obtained from SIGMA-ALDRICH, SANTA CRUZ BIOTECH(Dallas, TX), or AVANTI POLAR LIPIDS(Alabaster, AL). The standards were prepared in appropriate solutions and analyzed on gas chromatography time-of-flight mass spectrometry (GC-TOFMS) to establish in-house metabolite database. Sample preparation and metabolite quantification were performed according to the methods described in^53–56^. The frozen tissue and stool samples (50 ~ 100 mg) were homogenized on ice in 500-μL of a mixture of chloroform, methanol and water (1:2:1, v/v/v). The samples were then centrifuged at 13,000 rpm for 10 min at 4 °C, and a 150-μL aliquot of the supernatant was transferred to a sampling vial. The deposit was rehomogenized with 500-μL of methanol followed by a second centrifugation. Another 150-μL aliquot of supernatant was added to the same vial for drying. The residue was reconstituted with 500-μL of acetonitrile and water (1:1). The residue was then derivatized with 80-μL of methoxyamine (15 mg/mL in pyridine) was added to the vial and kept at 30 °C for 90 min, followed by 10-μL retention index compounds (mixture of C10-C40, 50 μg/mL) and 80-μL BSTFA (1% TMCS) at 70 °C for 120 min for derivatization to take place.

A 1-μL aliquot of the derivatized solution was injected into an Agilent 7890 N gas chromatograph in splitless mode coupled with a time-of-flight mass spectrometry (LECO Corp., St. Joseph, MI) used for untargeted metabolomics profiling. Separation was achieved on a Rxi-5ms capillary column (Crossbond 5% diphenyl/95% dimethyl polysiloxane; Restek) using helium as the carrier gas at a constant flow rate of 1.0 mL/min. The GC oven temperature began at 80 °C for the first 2 min, then raised at a rate of 10 °C/min to 140 °C, then at 4 °C/min to 180 °C, 10 °C/min to 240 °C, and 25 °C/min to 290 °C, and maintained at 290 °C for 4.5 min. The temperature of injection, transfer interface, and ion source was set to 270, 270, and 220 °C, respectively. The mass spectra were obtained with electron impact ionization (70 eV) at full scan mode (m/z 30–600) and an acquisition rate of 25 spectra/s.

The acquired MS files were exported in NetCDF format by ChromaTOF software (v4.22, LECO Corp., St. Joseph, MI). CDF files were extracted using custom scripts in the MATLAB (v7.0, Mathworks, Natick, MA) for data pretreatment procedures such as baseline correction, de-noising, smoothing, alignment, time-window splitting, and multivariate curve resolution^57^. Internal standards and any known artificial peaks, such as peaks caused by noise, column bleed and BSTFA derivatization procedure, were removed from the data set. Variance stabilizing normalization was performed on the resulting output data. Metabolite annotation was performed by comparing with reference standards in our in-house library. Commercially available mass spectral databases such as NIST library 2010 and LECO/Fiehn Metabolomics Library was also used for additional compound annotation (with a similarity threshed of 70%). Metabolomics data transformation was performed with variance stabilizing normalization. Unsupervised consensus hierarchical clustering was performed using ConsensusClusterPlus (v 1.38.0)^58^ with 10,000 iterations and 80% sample resampling from 2 to 10 clusters. The clustering result was visualized as a heatmap representation generated using ComplexHeatmap(v1.12.0)^59^. Statistical comparison between treatment and control was performed with exact Wilcoxon-Mann-Whitney test (at α = 0.05).

### Analysis of RNA expression profiles using public databases

We analyzed RNA expression profiles of CCA patients from the Oncomine database and The Cancer Genome Atlas (TCGA). Level 3 RNA data (unc.edu_CHOL.IlluminaHiSeq_RNASeqV2 Level 3.1.0.0) were obtained from TCGA database. The expression profile of each patient was median-centered, log2-transformed and loaded into MySQL database. RNA expression levels of tumor tissue and normal bile duct tissue from the same patient were extracted and analyzed. Gene expression profiles of the Woo Liver dataset from the Oncomine database (www.oncomine.org) were examined and compared to our data.

## Supplementary information

Supplementary Figure 1.
