# Supplementary material for: Long-term Proton Pump Inhibitor Administration Caused Physiological and Microbiota Changes in Rats
Source: Sci Rep. 2020 Jan 21;10:866. doi: 10.1038/s41598-020-57612-8 (PMC6972906; doi:10.1038/s41598-020-57612-8)
Supplement: Supplementary file 1 — Supplementary Figure 1. [file 41598_2020_57612_MOESM1_ESM.docx]

Supplementary information

**Long-term Proton Pump Inhibitor Administration Caused Physiological and Microbiota Changes in Rats**

Yu-Chen SH Yang^1#^, Hsuen-Wen Chang^2#^, I-Hsuan Lin^3^, Li-Nien Chien^4^, Min-Ju Wu^5^, Yun-Ru Liu^1^, Peiguo G. Chu^6^, Guoxiang Xie^7^, Fangcong Dong^7^, Wei Jia^7^, Vincent HS Chang^5*^, Yun Yen^3, 8, 9, 10, 11*^

^1^Joint Biobank, Office of Human Research, Taipei Medical University, Taipei, Taiwan

^2^Laboratory Animal Center, Office of Research and Development, Taipei Medical University, Taipei, Taiwan

^3^TMU Research Center of Cancer Translational Medicine, Taipei Medical University, Taipei, Taiwan

^4^School of Health Care Administration, College of Management, Taipei Medical University, Taipei, Taiwan

^5^The PhD Program for Translational Medicine, College of Medical Science and Technology, Taipei Medical University, Taiwan

^6^Department of Pathology, City of Hope National Medical Center, Duarte, CA 91010, USA

^7^University of Hawaii Cancer Center, Honolulu, Hawaii 96815, USA

^8^The PhD Program for Cancer Biology and Drug Discovery, College of Medical Science and Technology, Taipei Medical University, Taipei, Taiwan.

^9^Division of Chemistry and Chemical Engineering, California Institute of Technology, Pasadena, CA, 91125, USA

^10^Graduate Institute of Cancer Biology and Drug Discovery, TMU

^11^Cancer Center, Taipei Municipal WanFang Hospital

#These authors contributed equally to this work

*corresponding authors

Dr. Yun Yen.

The PhD Program for Cancer Biology and Drug Discovery, College of Medical Science and Technology, Taipei Medical University, Taipei, Taiwan; Phone: 886-2-27361661 ext.2008; Fax: 886-2-23787795; E-mail: [[yyen@tmu.edu.tw](mailto:yyen@tmu.edu.tw)] and [yunyen@caltech.edu].

Dr. Vincent HS Chang.

Departmentof Physiology, School of Medicine, College of Medicine, Taipei Medical University, Taipei, Taiwan; Phone: 886-2-27361661 ext. 7503; E-mail: [[vinhschang@tmu.edu.tw](mailto:vinhschang@tmu.edu.tw)].

Supplementary Figure 1


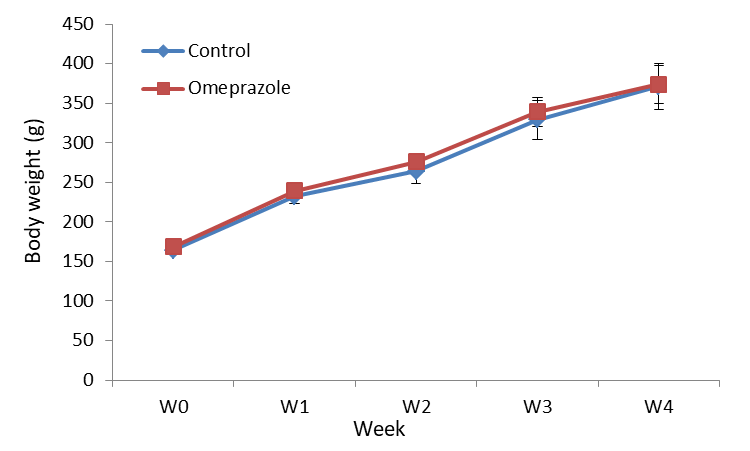


Supplementary Figure 1 : **The body weights of rat treated with omeprazole.** The body weights of rats in treatment and control groups were measured before and during treatment each week for 30 days. Long-term omeprazole treatment did not change body weights.
